# Small Pox

**Published:** 1854-01

**Authors:** 


*Small Pox.*—Mr. Marson, resident surgeon to the small pox and vaccination hospital, London, has published an "Analytic Examination" of the cases which occurred during four epidemics of this disease in the years 1838, 1844, 1848, 1851; of these, 3,094, rather more than one-half, had been vaccinated at some period in their lives. We have only room for the author's conclusions, which were.

1st. That natural small pox destroyed about one-third of all whom it attacked.

2d. That small pox after small pox, was of comparatively rare occurrence; that a second attack of natural small pox was rare, but not often fatal, and that protection seemed to be the law. That after inoculated small pox, an attack of small pox had more frequently led to fatal results; but there is reason to presume that the virus used for inoculation, like a great deal of the lymph used at the present day for vaccination, was often taken at too advanced a period of the disease, and thus did not afford the full measure of protection it was capable of affording, if taken at a proper time.

3d. That vaccination performed in infancy afforded almost complete protection against the fatality of small pox, to the period of puberty; that a variety of circumstances conspired to make it almost impossible to ascertain exactly in what proportion to the vaccinated cases of small pox subsequently occurred, or might occur, if all persons lived to an advanced age.

4th. That, as a matter of safety, it would be well for all persons who were vaccinated in infancy to be re-vaccinated at puberty; this measure being more especially requisite for those who were either indifferently or doubtfully vaccinated in infancy, and still more necessary for those who, though vaccinated, had no cicatrix remaining. Finally, as a matter of precaution, it would be desirable that all persons should be re-vaccinated, on small pox existing in the house where they were residing.

Dr. Chowne alluded to the neglects of vaccination in the country districts, which he stated to be a fault "of the government, by whom no efficient arrangements for vaccination was made, and consequently thousands lost their lives."

Mr. Marson stated that, "amongst the persons who had only been vaccinated in one place, and the cicatrix was imperfect, twenty per cent. took the small pox; whereas, when there were four cicatrices, and those were good, the number who took small pox, after vaccination, was only one per cent."—*From the N. H. Journal of Medicine.*

